# Venezuelan Equine Encephalitis Virus in Iquitos, Peru: Urban Transmission of a Sylvatic Strain

**DOI:** 10.1371/journal.pntd.0000349

**Published:** 2008-12-16

**Authors:** Amy C. Morrison, Brett M. Forshey, Desiree Notyce, Helvio Astete, Victor Lopez, Claudio Rocha, Rebecca Carrion, Cristhiam Carey, Dominique Eza, Joel M. Montgomery, Tadeusz J. Kochel

**Affiliations:** 1 Naval Medical Research Center Detachment, Iquitos and Lima, Peru; 2 Department of Entomology, University of California, Davis, California, United States of America; 3 Uniformed Services University of the Health Sciences, Bethesda, Maryland, United States of America; 4 Dirección Ejecutiva de Epidemiología de Salud de Loreto, Iquitos, Peru; 5 Centers for Disease Control, Atlanta, Georgia, United States of America; George Washington University, United States of America

## Abstract

Enzootic strains of Venezuelan equine encephalitis virus (VEEV) have been isolated from febrile patients in the Peruvian Amazon Basin at low but consistent levels since the early 1990s. Through a clinic-based febrile surveillance program, we detected an outbreak of VEEV infections in Iquitos, Peru, in the first half of 2006. The majority of these patients resided within urban areas of Iquitos, with no report of recent travel outside the city. To characterize the risk factors for VEEV infection within the city, an antibody prevalence study was carried out in a geographically stratified sample of urban areas of Iquitos. Additionally, entomological surveys were conducted to determine if previously incriminated vectors of enzootic VEEV were present within the city. We found that greater than 23% of Iquitos residents carried neutralizing antibodies against VEEV, with significant associations between increased antibody prevalence and age, occupation, mosquito net use, and overnight travel. Furthermore, potential vector mosquitoes were widely distributed across the city. Our results suggest that while VEEV infection is more common in rural areas, transmission also occurs within urban areas of Iquitos, and that further studies are warranted to identify the precise vectors and reservoirs involved in urban VEEV transmission.

## Author Summary

Venezuelan equine encephalitis (VEE) is a mosquito-borne viral disease often causing grave illness and large outbreaks of disease in South America. In Iquitos, Peru, a city of 350,000 situated in the Amazon forest, we normally observe 10–14 VEE cases per year associated with people traveling to rural areas where strains VEE virus circulate among forest mosquitoes and rodents. In 2006 we detected a 5-fold increase in human VEE cases, and many of these patients had no travel history outside the city where they lived. In response to this outbreak, we decided to determine if potential carrier mosquitoes were present within the city and if city residents had been previously exposed to the virus. We found that mosquitoes previously shown to transmit the virus in other locations were present—in varying amounts based on location and time of year—throughout Iquitos. A large percentage of the human population (>23%) had antibodies indicating past exposure to the virus. Previous VEE infection was associated with age, occupation, mosquito exposure, and overnight travel. Our data represent evidence of transmission of a forest strain of VEE within a large urban area. Continued monitoring of this situation will shed light on mechanisms of virus emergence.

## Introduction

Members of the Venezuelan equine encephalitis virus (VEEV) complex are arboviruses belonging to the *Alphavirus* genus of the *Togaviridae* family. First identified among equines in the 1930s [1], VEEV-associated human disease was not recognized until 1943 [2],[3] , although epidemiological data suggest that outbreaks may date back to the 1920s [4]. VEEV subtypes cause a wide clinical spectrum of disease ranging from undifferentiated fever to severe neurological symptoms, with a case fatality rate of 1–4% [5]. Two transmission cycles have been identified: an enzootic cycle, maintained among rodent reservoirs in forest habitats, and an epizootic cycle that causes high rates of mortality in horses as well as epidemics among human populations [4]. These cycles are typically associated with distinct subtypes of the VEE virus complex: subtypes IAB and IC with equine epizootics, subtypes ID, IF, and II–VI with the equine avirulent enzootic cycle [4],[6], and subtype IE with both enzootic and equine-virulent transmission cycles [7],[8],[9]. Despite disparate serological and clinical phenotypes some enzootic and epizootic subtypes are highly genetically conserved. In particular, strains of the enzootic subtype ID (Columbia/Venezuela genotype) show less than 0.5% divergence from epizootic IAB and IC subtypes at the amino acid level [10],[11],[12]. Based on this genetic conservation, epizootic strains have been proposed to emerge periodically from progenitor strains continuously maintained in an enzootic forest cycle. Accordingly, a single amino acid change within the E2 envelope gene has been shown to confer an epizootic phenotype on an enzootic VEEV strain [10],[11],[12],[13].

Geographically, members of the VEEV antigenic complex have been restricted to tropical and sub-tropical regions of the Western Hemisphere, with VEEV complex isolates reported from Argentina through the southern United States. The majority of human VEEV infections have occurred during large outbreaks in Central America and northern South America, most notably in Colombia and Venezuela [14],[15]. In Peru, multiple human epidemics and equine epizootics have occurred on the Pacific coastal plain, possibly due to introduction of epizootic virus from Ecuador. At present, all evidence suggests that the epidemiology of VEEV on the west coast of Peru has been associated exclusively with the epizootic subtype IAB [3],[16],[17],[18]. In contrast, in the Amazon Basin to the east of the Andean mountains VEEV isolates have been restricted to enzootic ID, IIIC, and IID subtypes. The first cases of human VEEV infection in this region were reported in 1994 when Peruvian Army personnel were deployed to an area near Iquitos [18],[19]. Human VEEV cases have been documented near Iquitos continuously since then [6],[19] (TJK, unpublished data). Based on entomological studies carried out in the nearby village of Puerto Almendras and the Otorongo Military Base from 1996–2001 [20], mosquitoes from the *Culex (Melanoconion)* group have been incriminated as the local sylvatic VEEV vector in rural areas, consistent with results from Panama, Colombia, and Venezuela [4],[5],[21],[22],[23]. While potential vectors of enzootic VEEV have also been periodically detected within urban neighborhoods [24],[25], the possibility for urban transmission of enzootic VEEV has not been systematically addressed. The city of Iquitos represents the interface between the Amazon forest and a densely populated urban environment, and therefore a potential bridge between enzootic transmission cycles and potential peridomestic urban transmission.

In 1990, the Naval Medical Research Center Detachment (NMRCD) initiated a clinic-based surveillance program to determine the etiologies of febrile illness within Iquitos as well as nearby villages. Herein we report evidence of a 2006 outbreak of febrile illness associated with enzootic VEEV infection detected by the NMRCD surveillance program. Following the outbreak, a seroprevalence survey was carried out in three Iquitos neighborhoods where acute human cases were identified as well as in a control neighborhood where acute cases were not reported during the 2006 outbreak. Additionally, a series of mosquito collections were conducted both during and after the outbreak to characterize potential urban VEEV vectors. The primary objective of this article is to evaluate the evidence for peri-domestic VEEV transmission within the city of Iquitos, Peru.

## Materials and Methods

### Study Site

The study was conducted in the Loreto Department in Peru in the city of Iquitos located 120 meters above sea level in the Amazon forest (73.2°W, 3.7S°). This site has been described in detail previously [24],[25],[26],[27],[28]. Briefly, Iquitos is a geographically isolated population within the Amazon forest, accessible only by river or air travel. The major industries of Iquitos are small business, fishing, oil, lumber, tourism and some agriculture [27]. The climate is tropical, with an average daily temperature of 25°C and year-round precipitation totaling 2.7 meters. Daily temperature and precipitation data for 2000–2006 from a weather station located at the Iquitos airport were retrieved from http://lwf.ncdc.noaa.gov/cgi-bin/res40.pl?page=climvisgsod.html (Figure S1). River levels surrounding the city change dramatically due to runoff from the eastern side of the Andes mountains, increasing by up to 10 m (108–118 m) between the “vaciente” (May–November) and “creciente” (December–April) [29]. Information for daily river levels was obtained from the local water plant (Figure S1).

**Figure 1:**
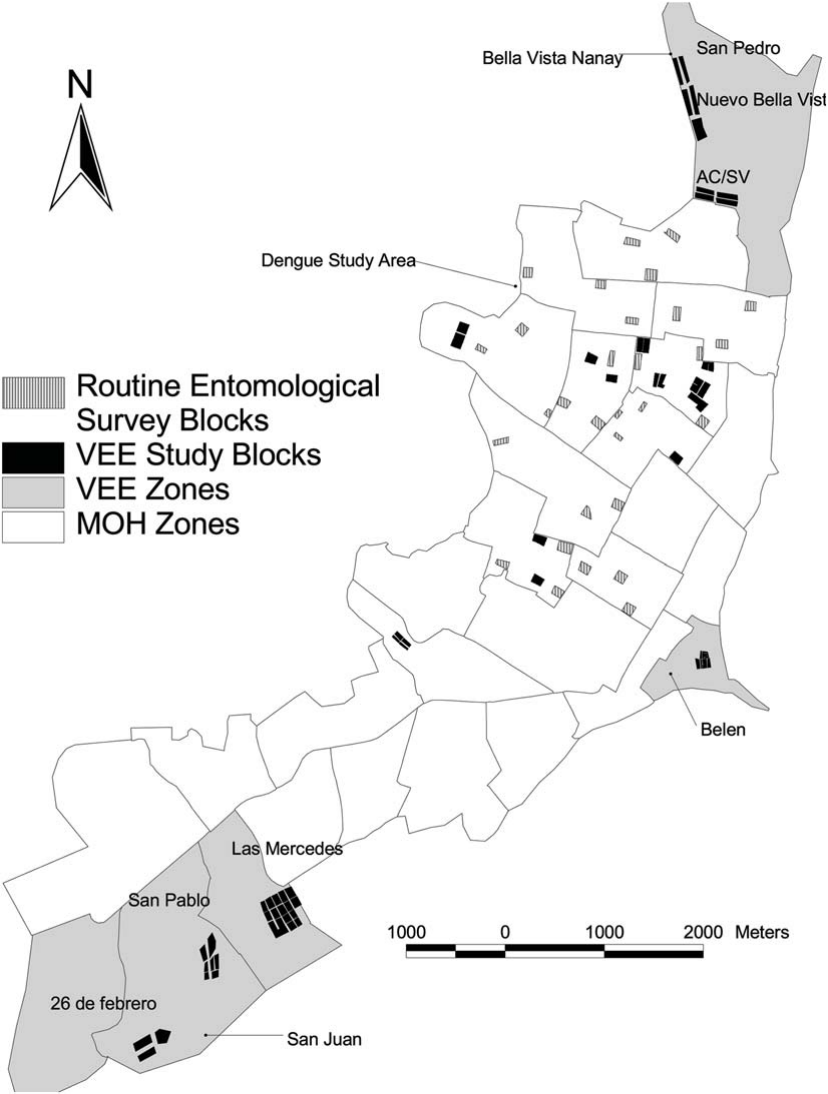
Map of Iquitos showing the locations of the 4 major study areas. The neighborhoods where active VEE cases were identified are shown in grey and the study blocks are shown in black. Study blocks in the dengue study area (white background) are also shown in black. The blocks where routine entomological surveys were carried out between January 2006 and January 2007 are shown in black and white stripes.

Serological and entomological surveys described in the study were initially targeted to three areas of Iquitos based on the residences of VEEV-infected patients detected through a clinic-based surveillance system during 2006 (Figure 1). The neighborhoods included in this study were Bella Vista Nanay (San Pedro, Nuevo Bellavista, Acción Católica, Nuevo Amanecer, and San Valentín) located in the northern-most section of the city; Belén, located in the eastern part of the city along the Itaya River; and three sites along Avenida Participación in the San Juan District (Las Mercedes, San Pablo de la Luz, and 26 de Febrero). Common attributes of the Bella Vista, Belén, and San Juan sites include seasonal flooding, and proximity to rivers, lowland humid tropical forest, and open farmland. The area surrounding Iquitos has experienced varying degrees of deforestation, but patches of both primary and secondary growth trees are found on opposite banks of the three surrounding rivers. The habitat observed in all three neighborhoods is rather homogeneous. Species diversity, including a wide variety of aquatic plants (*Onagraceae*, *Pontederiaceae*, *Araceae* families) and abundance was highest in the Avenida Participación neighborhoods, followed by Bella Vista Nanay, and finally Belen which had the highest density of housing and port activity (Table S1 for species list). The Bella Vista site is seasonally flooded by the blackwater Nanay River, whereas the Belen and San Juan sites are located on the silty and sediment-rich whitewater Itaya River. The Allpahuayo National Reserve and “Ell Huayo” Botanic Garden are located ∼25 km to the south of the city where both feral mammalian and forest mosquito species have been well characterized [30],[31].

In addition to the three neighborhoods with active VEEV cases during 2006, we obtained blood samples from residents in designated a control area where no increase in VEEV activity was detected. This area included 22 blocks in north central parts of Iquitos where previous dengue studies had been conducted (ACM, unpublished data). This geographically diverse group of blocks was easily accessible to our research team because of our previous studies there, and represented a contrast to the 3 study neighborhoods. Overall, these control neighborhoods were of higher socio-economic condition [29] (Morrison unpublished data) and located several blocks from the river whereas the other study areas were adjacent to the river. Municipal records obtained from “La asociación de viviendas inundables y desarrollo humano de Punchana” and “El Programa de Emergencia Social Productivo Urbano” indicate that the neighborhoods in BellaVista Nanay and Avenida Participación (“New”) were all established since 1998–2003 whereas the Belen and control blocks (“Old”) have been registered since 1943 and have existed prior to that date.

### Study Design

We will present data from 3 separate studies. First we will describe a bimodal outbreak of febrile illness attributed to VEEV infection that began in 2005 and culminated with a significant increase in cases in the first half of 2006. This notable increase in human cases stimulated NMRCD to carry out a cross-sectional seroprevalence study in three neighborhoods where VEEV cases had been detected within the city limits of Iquitos, as well as a series of entomological studies to document the abundance of and VEEV infection rates in potential mosquito vectors. Below we describe in detail the methods associated with each sub study.

### Febrile Surveillance Study

Since 2000, NMRCD has been conducting syndromic surveillance in 11 Government Health Centers and Hospitals (9 urban and 2 rural). This study, entitled “Surveillance and Etiology of Acute Febrile Illness,” was approved by the NMRCD Institutional Review Board (NMRCD.2000.0006). Trained health workers are stationed in each location 0700-1300. All acute, undifferentiated, febrile illness cases (i.e. temperature greater than or equal to 38°C for 7 days duration or less) seen by a health center physician were referred first to the national Malaria program where they are tested for Malaria by a thick smear and then to our surveillance program. For inclusion into the study, in addition to fever, patients needed to report one or more of the following symptoms: headache; muscle, ocular and/or joint pain; generalized fatigue; cough; nausea; vomiting; sore throat; rhinorrhea; difficulty breathing; diarrhea; bloody stools; jaundice; dizziness; disorientation; stiff neck; petecchiae; ecchymoses; bleeding gums or nose. Children younger than five years of age were included if they presented with hemorrhagic manifestations indicative of dengue hemorrhagic fever (DHF), including bleeding gums or nose, petecchiae, bloody stool or hematemesis. Written informed consent was obtained from adults greater than 18 years of age. For minors, written consent was obtained from parents, and assent was obtained from participants ages 8–17. For participants unable to read and sign the consent form, a witness was present to testify to oral consent. Demographic data, residential address, medical history, and clinical features for each patient were obtained using a standard questionnaire. During the acute phase of illness blood samples were obtained from each patient, and when possible, convalescent samples were obtained 10 days to 4 weeks later for serological studies. In addition, axial temperature, blood pressure, and respiratory rate were recorded, and in most facilities a tourniquet test was performed. Exclusion criteria included a clear focus of infection (i.e. respiratory, gastrointestinal, urinary tract).

Acute and convalescent samples were tested for a range of arboviruses including VEEV. Diagnoses were considered confirmed if they met the following criteria: clinical diagnosis along with laboratory confirmation (isolation of virus from the sample, identification by RT-PCR, or 4-fold increase in IgM antibody titers). Patients' residences were located using existing GIS data for Iquitos [26] and confirmed by study team members.

### Serological Surveys

In response to the notable increase in VEE cases observed in early 2006, serological surveys were initiated in three neighborhoods with high VEEV activity between January–June 2006 and on blocks that had participated in a previous dengue cohort study (22 city blocks located in 7 geographic zones) located in the districts of Maynas, San Juan and Punchana. Surveys were conducted between early November and mid-December of 2006. The post-outbreak study protocol was reviewed and approved by the NMRCD Institutional Review Board (PJT.NMRCD.001).

Trained phlebotomists (10–22 in total) working in two person teams were assigned individual maps and proceeded door to door to explain the study and recruit participants. Participation was offered to all individuals ≥5 years of age. If the residents agreed to participate, the consent and assent forms were signed before samples were obtained. Written informed consent was obtained from participants older than 18 years, and from parents of participants younger than 18. In addition, assent was obtained from participants 8–17 years of age. If participants were unable to read and sign the consent form, oral consent was obtained and documented in the presence of a witness. Each participant was asked a series of questions about their homes, as well as travel histories and illnesses during the previous year. Younger children (<14 years) were interviewed with their parents. Blood samples were obtained using standard aseptic techniques using a vacutainer tube and 21–23 gauge needles. All blood samples were tested for anti-VEEV antibodies using IgG and IgM ELISAs. All samples that tested positive by IgG ELISA were further evaluated for anti-VEEV antibodies by the plaque reduction-neutralizing test (PRNT).

### Entomological survey

Two adult mosquito collection methods were used for this study. First, standard household surveys were carried out as previously described [24],[25]. In these surveys adult mosquitoes were collected using a backpack aspirator both inside and outside the house. Mosquito collections were concentrated in areas with high numbers of human VEEV cases during the 2006 outbreak. Second, CDC light traps baited with CO_2_ were placed outdoors between 1800-0600 h on four continuous nights in Bella Vista Nanay, Belen and in San Juan neighborhoods of Las Mercedes, San Pablo de la Luz and 26 de Febrero. Adult mosquitoes were identified to species [32] on dry ice and sorted into plastic vials by species for storage at −70°C for later testing for VEEV RNA by RT-PCR.

### Laboratory Assays

#### Virus isolation and identification

Acute-phase serum samples from febrile patients were diluted 1∶5 in minimum essential medium containing 2% heat-inactivated fetal bovine serum and antibiotics and subsequently inoculated onto African Green Monkey Vero (37°C) and mosquito (*Aedes albopictus*) C6/36 (28°C) cell cultures [33]. Upon observation of viral cytopathic effect, or ten days post-inoculation if no CPE was observed, cells were removed from the flasks and placed on 12-well glass spot-slides for examination by immunofluoresence assay (IFA) using anti-VEEV polyclonal antibodies. Viral RNA was extracted from VEEV-positive cultures, amplified by reverse transcriptase-PCR (RT-PCR) and sequenced, as described elsewhere [6],[34].

#### IgG and IgM ELISA

Participants' sera were assayed for IgG and IgM antibodies against VEEV antigen (VEEV VR69 ATCC) by ELISA as previously described using a VEEV (vaccine strain TC-83 subtype IAB) lysate antigen from Vero cells [35],[36]. The positive cutoff optical density (OD) value was calculated from the mean adjusted OD of antibody negative control sera plus 3 standard deviations.

#### Plaque Reduction Neutralization Test (PRNT)

PRNT protocol was modified from Morens et al. (1985) [37]. Briefly, participants serum was heat-inactivated at 56°C in a water bath for 30 minutes and diluted in media (Eagle's Minimal Essential Medium [EMEM] + Penicillin/Streptomycin [Pen./Strep]) from 1∶10 to 1∶500. VEEV (subtype IAB vaccine strain TC83 TVB5215) was diluted to contain 15 plaques/well in 24-well TC-plate. The viral dilution was mixed in equal volume of the participant's serum and incubated at 4°C overnight. The serum-virus mixture was subsequently inoculated onto a monolayer of Vero-76 cells in 24-well plates (seeded with 0.5 ml/well of 3×10^5^ cells/ml) and incubated at 37°C in 5% CO_2_ for 3 hours. 0.5 ml overlay media (0.6% Carboxymethyl Cellulose, Minimum Essential Medium [MEM] without Phenol Red, 10% Fetal Bovine Serum [FBS], 0.075% NaHCO_3_ and Pen/Strep) was added to the wells and incubated for 3 days. On day three media was removed, and the cells rinsed with H_2_O and stained with 0.5 ml/well stain solution (0.1% (w/v) Naphthol Blue Black, 1.36% (w/v) Sodium Acetate, and 6% (v/v) Glacial Acetic Acid) for 30 min. The stain was then removed and the plaques where counted. The results were expressed as the serum dilution, determined by probit analysis that reduced the number of plaques by 70% compared to that of normal human serum at the same dilution. The conditions used in determining PRNT titers (70% plaque reduction and inverse titer cutoff of 60) were based on experiments performed to optimize sensitivity and specificity of the VEEV PRNT in our laboratory. The potential for cross-reaction due to prior infection by co-circulating alphaviruses, such as Mayaro virus, necessitated a higher cutoff level than used elsewhere [38].

#### RT-PCR analysis of mosquito homogenates

Mosquitoes collected by CDC light traps were tested for VEEV RNA using RT-PCR modified from Lanciotti *et al.* (2000) [39]. Briefly, mosquitoes were pooled by species and date collected and subsequently homogenized. Viral RNA was then extracted and purified using QIAamp viral RNA kit (QIAGEN, Valencia, Calif.). Primers sequences were specific for VEEV subtypes ID and III [6],[34]: V8369 (GAGAACTGCGAGCAATGGTCA) and V9207B(-) (TRCACTGGCTGAACTGTT) for subtype ID, and V8369 and V9257B(-) (TACACCCAYTTRTCRTTCTG) for subtype III.

### Statistical analysis

Proportions were compared using a chi-square test using the FREQ procedure in SAS (SAS Version 8, 1999, SAS Institute Inc., Cary, NC.). Risk factors for infection with VEEV were evaluated by logistic regression using LOGISTIC in SAS. Models were constructed with the dichotomous dependent variable: PRNT positive for VEEV antibody at a titer of ≥1∶60 and the following independent variables: age (adult, child), occupation, travel history (report of multiple day trips outside Iquitos), and animals (on property).

## Results

### Evidence for VEE Outbreak

From 2000–2004 the NMRCD febrile surveillance program detected up to four VEEV cases per month with annual totals ranging from 10–14 cases (Table 1). In 2005, however, 15 cases were identified in June and July, with an annual total of 27. Fifteen of these cases came from rural clinics Zungaracocha (10 cases) and Quistococha (5 cases). An additional five cases came from Hospital Apoyo, which serves patients from the entire Department of Loreto; three of these five maintained residence outside of Iquitos. In 2006, there were 63 confirmed cases of VEEV infection captured in the febrile surveillance study (Table 1), representing a 5-fold increase in the number of cases from the 2000–2004 average. Of these 63 cases, 29 were identified by IgM seroconversion, and 34 were identified by IFA and RT-PCR. The partial PE2 nucleotide sequence was determined for a subset of the viral isolates and compared to previously characterized VEEV strains; all sequenced isolates were found to belong to the enzootic Panama/Peru ID subtype (Figure S2), closely related to previous isolates from the region [6].

**Table 1:** Laboratory confirmed cases of VEEV infection identified in 11 health facilities in or near Iquitos, Peru.

Health Center	Surveillance Year							Total
	2000	2001	2002	2003	2004	2005	2006	
Bella Vista Nanay	0	1	7	0	1	1	10	**20**
San Antonio	0	0	0	0	2	4	0	**6**
Clinical Naval	-	1	0	0	0	1	3	**5**
Hosp. Apoyo Iquitos	1	0	0	0	4	5	8	**18**
Santa Rosa	-	2	0	1	0	0	0	**3**
Moronacocha	0	1	0	1	1	1	6	**10**
Tupac Amaru	0	2	6	0	1	0	5	**14**
Belen	1	1	1	2	1	0	17	**23**
San Juan	8	0	0	8	0	0	5	**21**
**Quistococha** *	-	-	-	-	1	5	6	**12**
**Zungarococha**	1	2	0	2	3	10	3	**21**
**Total**	**11**	**10**	**14**	**14**	**14**	**27**	**63**	**153**

Note: centers listed in bold are located in rural areas outside Iquitos.

*: Site was activated in 2004.

Of the 63 cases detected in 2006, 60 were detected from February to July, with the peak occurring in April and May. In both 2005 and 2006, VEEV activity was concentrated during the first half of the year, as river levels were increasing to a peak in April and May. Precipitation levels were higher during January–March in both 2005 and 2006 when compared to 2000–2004 and in 2006 river levels were higher than previous years. Sixty of the VEEV-infected individuals reported to public health centers, and three were Peruvian Navy personnel reporting to a military health center. Health facilities in Belen, Bella Vista Nanay and San Juan were the urban centers with the highest 6-year and 2006 VEEV case totals (Table 1). The demographic information and travel history of the civilian cases observed in 2006 are summarized in Table 2, with comparison to other febrile patients reporting to public health centers during the same year. No statistically significant differences were found between VEEV patients and other patients in gender, travel history, or occupation (data not shown). Compared with other febrile patients, a higher percent of VEEV patients reported residences outside of urban Iquitos (Table 2; χ^2^ = 10.2, *df* = 1, P<.005). Despite this bias, the majority of VEEV patients resided within the city (44, 73.3%) and did not report history of travel within the 30 days preceding their illness (53, 88.3%).

**Table 2:** Demographic information of VEEV febrile patients and all other febrile patients reporting to public health centers in 2006.

Characteristic	Subcategory	VEEV Cases	All Febrile Cases
No. Acute Cases		60	1136
Sex	Male	32 (53.3%)	531 (46.7%)
	Female	28 (46.7%)	605 (53.3%)
Median Age (Range)		23.5 (6–71)	24 (1–82)
Recent Travel		7 (11.7%)	197 (17.6%)
Residence outside Iquitos		16 (26.7%)	141 (12.4%)
Occupation	Student	19 (31.7%)	349 (30.7%)
	Housewife/Work from Home	16 (22.3%)	335 (29.5%)
	Vendor/Merchant	7 (11.7%)	97 (8.5%)
	Laborer	4 (6.7%)	43 (3.8%)
	Agriculture	3 (0.5%)	18 (1.6%)
	Other	11 (18.3%)	294 (25.8%)

### Seroprevalence Survey

#### Population sampled

In response to the 2006 VEEV outbreak, a seroprevalence survey was conducted in neighborhoods within Iquitos. A total of 1,327 participants were enrolled from November 2 to 30, 2006. Of these, 13 were not included in the analysis due to inconclusive serological results (insufficient sera for PRNT); 11 were participants from the control blocks and 1 each from Belen and Bella Vista Nanay. The mean age of the 1,314 evaluated subjects was 29 years (range 5–88 years); 70.7% (920) were adults (≥18 years old); and 67% (880) were female (Table 3). Most of the respondents (99.9%) claimed to be permanent residents of Iquitos, with only 17.5% (230) reporting multi-day trips outside Iquitos and 24.9% (327) reporting day excursions (Table 3). The most common occupation reported was housewife (561, 42.7%), followed by student (418, 31.8%) and merchant/vendor (107, 8.1%). Only 26 (1.8%) individuals reported occupations considered to be at high risk for exposure to a forest VEEV cycle (fisherman, or laborer working in agriculture, the petroleum industry, or logging/lumber).

**Table 3:** Characteristics of 1,314 subjects living in three neighborhoods reporting VEEV cases between January and June, 2006, and 22 blocks previously included in Dengue cohort studies.

Neighborhood	Gender (Males)	Age (<18 years)	Occupation (High Risk)	Travel (Yes)	Excursion (Yes)	Animals (Yes)	House (Noble)	Mosquito Net (Use)	Syndrome (Yes)
**Belen**	**126**	**97**	**13**	**57**	**78**	**193**	**34**	**253**	**113**
	**(36.5%)**	**(28.1%)**	**(3.8%)**	**(16.5%)**	**(22.6%)**	**(55.9%)**	**(9.9%)**	**(73.3%)**	**(32.8%)**
**Bella Vista Nanay**	**94**	**94**	**5**	**65**	**119**	**182**	**28**	**316**	**119**
	**(29.2%)**	**(29.2%)**	**(1.6%)**	**(20.2%)**	**(37.0%)**	**(56.5%)**	**(8.7%)**	**(98.1%)**	**(37.0%)**
San Pedro	32	26	3	27	40	52	11	89	34
	(35.6%)	(28.9%)	(3.3%)	(30.0%)	(44.4%)	(57.8%)	(12.2%)	(98.9%)	(37.8%)
Nuevo Bellavista	27	40	1	21	37	74	12	118	43
	(22.9%)	(33.9%)	(0.9%)	(17.8%)	(37.4%)	(62.7%)	(10.2%)	(100%)	(36.4%)
AC/SV/NA	35	28	1	17	43	56	5	110	42
	(30.4%)	(24.4%)	(0.9%)	(14.8%)	(37.4%)	(48.7%)	(4.4%)	(95.7%)	(36.5%)
**San Juan**	**109**	**97**	**7**	**65**	**64**	**247**	**68**	**334**	**132**
	**(31.8%)**	**(28.3%)**	**(2.0%)**	**(19.0%)**	**(18.7%)**	**(72.0%)**	**(19.8%)**	**(97.4%)**	**(38.5%)**
Las Mercedes	43	26	3	28	33	110	56	125	44
	(33.9%)	(20.5%)	(2.4%)	(22.1%)	(26.0%)	(86.6%)	(44.1%)	(98.4%)	(34.7%)
San Pablo	51	49	3	36	29	103	12	160	34
	(30.5%)	(29.3%)	(1.8%)	(21.6%)	(17.4%)	(61.7%)	(7.2%)	(95.8%)	(37.8%)
26 de Febrero	15	22	1	1	1	34	0	48	32
	(31.3%)	(45.8%)	(2.1%)	(2.1%)	(2.1%)	(70.8%)	(0.0%)	(100%)	(66.7%)
**Control**	**105**	**106**	**1**	**43**	**66**	**250**	**197**	**227**	**82**
	**(34.5%)**	**(34.9%)**	**(0.3%)**	**(14.1%)**	**(21.7%)**	**(82.2%)**	**(64.8%)**	**(74.7%)**	**(27.0%)**
**TOTAL**	**434**	**394**	**26**	**230**	**327**	**872**	**327**	**1130**	**446**
	**(33.0%)**	**(29.3%)**	**(1.8%)**	**(17.5%)**	**(24.9%)**	**(66.4%)**	**(24.9%)**	**(86%)**	**(33.9%)**

The 4 major neighborhoods are indicated in bold, whereas areas within each neighborhood are indicated in normal text.

Among the respondents, 33.9% reported fever, body aches and headache (listed as “syndrome” in Table 3) in the previous six months. Reports of febrile syndrome were highest in Bella Vista Nanay and San Juan (Table 3; χ^2^ = 11, *df* = 3, P = 0.0104). Eleven participants reported loss of consciousness in conjunction with febrile syndrome; three of these also reported convulsions during the previous six months.

#### Study population characteristics by neighborhood

Characteristics of the study population by neighborhood are shown in Table 3. Females participated at a higher rate than males, and adults more often than children; this pattern was consistent across neighborhoods (χ^2^ = 8.9, *df* = 7, P = 0.26; χ^2^ = 4.5, *df* = 3, P = 0.20, respectively) with the exception of the control blocks (106 of 304, 34.9%) which had a higher proportion of minors (<18 years; 288 of 1010, 28.5%; χ^2^ = 4.5, *df* = 1, P = 0.034). Participants reporting occupational exposure to forest environments were low overall. Belen had the highest (3.8%) proportion of participants in high exposure occupations whereas the control blocks had the lowest (0.3%;χ^2^ = 10.3, *df* = 3, P = 0.0164).

In the control blocks 64.8% of the houses were made with concrete or brick compared with 8.7% to 19.8% in the neighborhoods reporting active VEEV cases (χ^2^ = 351, *df* = 3, P<0.0001). Use of mosquito nets by residents was high overall (86%) but varied by neighborhood (χ^2^ = 154, *df* = 3, P<0.0001). Domestic animals (dogs, cats, chickens, and pigs) were more prevalent in the control areas (82.2%) than the VEEV blocks (61.6%) (χ^2^ = 44, *df* = 1, P<0.0001). In contrast, rodents were reported most often in households in Belen (95.7%) and Bella Vista Nanay (90.7%), followed by San Juan (82.8%) and control neighborhoods (79.6%) (χ^2^ = 48, *df* = 3, P<0.0001).

Participants were asked about trips and excursions they had taken in the previous 6-month period. The questions were designed to distinguish between longer (overnight trips) and short day trips to recreational areas near Iquitos. Participants reporting longer versus shorter trips ranged from 14–20% and 19–37% of the respondents, respectively. The majority of the trips reported were to river communities or recreation areas within the Amazon Basin.

#### Antibody prevalence analysis

Participant samples were initially screened by VEEV-specific IgG ELISA and subsequently confirmed by PRNT. Of the 1,314 participants included in the analysis, 71.8% (943) tested negative and 28.2% (371) tested positive for anti-VEEV antibodies by IgG ELISA (Table 4). Following the initial screening, 313 (23.8% of the total samples) of the 371 IgG-positive samples also had VEEV neutralizing antibodies at a titer of ≥1∶60. Of these, 28 had titers between 1∶60 and 1∶98, 122 were between 1∶101 and 1∶495, 118 were between 1∶500 and 1∶2402, and 45 were greater than 1∶2560. Of the 1136 samples, only three had positive IgM antibody titers against VEEV.

**Table 4:** Prevalence of anti-VEEV antibodies among 1,314 subjects living in 4 neighborhoods in Iquitos, Peru.

Neighborhood*	No. Samples	IgG (−)	IgG (+)		All (-)
			PRNT (−)	PRNT (+)	
**Belen**	**345**	**258 (74.8%)**	**26 (10.6%)**	**61 (17.7%)**	**284 (82.3%)**
**Bella Vista Nanay**	**323**	**201 (62.2%)**	**23 ( 7.1%)**	**99 (30.7%)**	**224 (69.3%)**
San Pedro	90	45 (50.0%)	8 (8.9%)	37 (41.1%)	53 (59.9%)
Nuevo Bellavista	118	66 (55.9%)	8 (6.8%)	44 (37.3%)	74 (62.7%)
AC/SV/NA	115	90 (78.3%)	7 (6.1%)	18 (15.7%)	97 (84.3%)
**San Juan**	**342**	**239 (69.9%)**	**9 ( 2.6%)**	**94 (27.5%)**	**248 (72.5%)**
Las Mercedes	127	91 (71.7%)	3 ( 2.4%)	33 (26.0%)	94 (74.0%)
San Pablo	167	118 (70.7%)	6 ( 3.6%)	43 (25.7%)	124 (74.3%)
26 de Febrero	48	30 (62.5%)	0 ( 0.0%)	18 (37.5%)	30 (62.5%)
**Control**	**304**	**245 (80.6%)**	**0 ( 0.0%)**	**59 (19.4%)**	**245 (80.5%)**
**Total**	**1,314**	**943 (71.8%)**	**58 (4.4%)**	**313 (23.8%)**	**1,001 (76.2%)**

*: Neighborhoods in bold print are shown in Figure 1 and represent totals for housing areas listed below. Belen, Bella Vista Nanay and San Juan neighborhoods reported VEEV cases between January–June, 2006 in clinic-based passive febrile surveillance studies. The control areas consist of 22 blocks previously included in Dengue cohort studies.

Antibody prevalence rates varied between neighborhoods, but were highest in San Pedro and Nuevo Bellavista located in Bella Vista Nanay (37–41%) and 26 de Febrero (38%) located in San Juan, followed by Las Mercedes and San Pablo in San Juan (26–30%; Table 4). Despite the high number of VEEV cases reported from Belen in 2006, antibody prevalence rates were similar to those in the control area, which had reported fewer VEEV cases that year. The neighborhoods in Belen and the control neighborhoods are older than those in San Juan and Bella Vista Nanay. Thus, the major contrast was between older neighborhoods where 18.5% of participants had antibody to VEEV compared to newer neighborhoods with rates of 29% (χ^2^ = 20, *df* = 1, P<0.0001).

#### Risk factors

**Figure 2:**
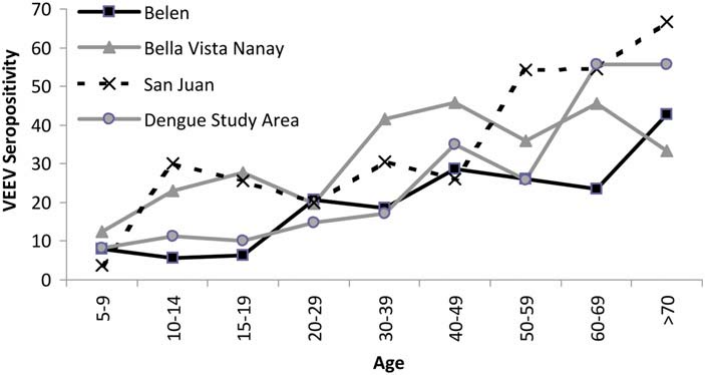
Age-dependent increase of VEEV-neutralizing antibodies.

Gender, day travel, presence of domestic animals, rodent sightings on the property, and the type of house construction materials had no association with VEEV antibody prevalence in our study sample (Table 5). Variables associated with VEEV antibody positivity in univariate logistic regression analysis included age, occupation, extended overnight travel, mosquito net use, and history of febrile illness syndrome (fever, body aches, headache) in the previous six-month period (Table 5). VEEV antibody positivity increased with age, with clear neighborhood differences (Figure 2). Antibody prevalence rates were higher among 10–29 year-old subjects in the BellaVista Nanay and San Juan neighborhoods (34%), compared to the Belen and control neighborhoods (24%; χ^2^ = 8.6, *df* = 1, P = 0.0033). Even though only 26 sample participants reported a high-risk occupation, this group had higher odds of VEEV infection than all other occupations combined (OR = 4.5, 95% CI 2.1, 10.0). Individuals reporting overnight travel outside Iquitos also had higher odds VEEV infection, but, interestingly, not those reporting day trips. It is probable that there was some confusion between the two questions for respondents. The Spanish terms “Viaje” (trip) and “Paseo” (excursion) are often interchangeable. To address this, we combined both categories into a single variable; in combination, travel was not significantly associated with VEEV antibody response (Table 5), indicating risk was only associated with overnight travel further from the city . Mosquito net use and history of febrile illness were both associated with previous VEEV infection. There were more reports of febrile illness in the areas with higher antibody prevalence rates (Table 5), perhaps reflecting the recent VEEV activity detected in the passive febrile surveillance system.

Multivariate logistic regression was performed, evaluating all demographic and potential risk factor variables described above in a stepwise fashion with VEEV antibody prevalence as an outcome variable. The neighborhood variable was collapsed into older (Belen and control blocks) and newer (Bella Vista Nanay and San Juan) areas. All risk factors identified in the univariate analysis remained in the final model. The adjusted odds ratios are shown in Table 6.

**Table 5:** VEEV antibody prevalence by potential risk factors for infection and odds ratio in univariate logistic regression.

Variable	Category	Percent Prevalence (No. Positive/No Tested)	OR (95% CI)
Gender	Female	23.2 (204/880)	0.90
	Male	25.1 (109/434)	(0.69–1.18)
Age	<18 years	14.7 (58/394)	2.22
	≥18 years	27.7 (255/920)*	(1.62–3.04)
Occupation	High Risk	57.7 (15/26)*	4.53
	Low Risk	23.1 (298/1288)	(2.06–9.97)
Neighborhood	Old (Belen/Control)	18.5 (120/649)	1.80
	New (BellaVista/San Juan)	29.0 (193/665)*	(1.39–2.34)
Travel (Multi-day)	Yes	31.3 (72/230)**	1.59
	No	22.2 (241/1084)	(1.17–2.18)
Travel (Day trip)	Yes	22.9 (75/327)	0.94
	No	24.1 (238/987)	(0.64–1.26)
Travel (Multi or 1 day)	Yes	26.6 (130/488)	1.28
	No	22.2 (183/826)	(0.98–1.65)
Animal	Yes	25.1 (219/872)	1.24
	No	21.3 (94/442)	(0.94–1.63)
Rodent Sightings	Yes	25.3 (271/1148)	0.91
	No	23.6 (42/166)	(0.63–1.33)
Mosquito Net	Use	25.5 (288/1130)**	2.18
	Don't Use	13.6 (25/184)	(1.40–3.39)
Housing Materials	Wood	21.1 (69/327)	0.81
	Concrete/Brick	24.7 (244/987)	(0.60–1.10)
Febrile Syndrome	Yes	29.2 (130/446)**	1.54
	No	21.1 (183/868)	(1.86–2.00)

*: *P<0.0001* by χ^2^ test.

**: *P<0.005* by χ^2^ test.

**Table 6:** Adjusted odds ratios of potential risk factors associated with VEEV antibody status, based on a stepwise multivariate logistic regression model.

Independent Variables	Odds Ratio	95% CI
Age (referent, child)	2.13	1.54–2.93
Travel (referent, no history of travel)	1.43	1.03–1.98
Neighborhood (referent, older neighborhoods)	1.53	1.16–2.02
Occupation (referent, low risk)	3.44	1.52–7.80
Febrile Syndrome (referent, none)	1.46	1.12–1.92
Mosquito Net (referent, Don't Use)	1.88	1.17–3.02

### Entomological Surveys

#### Backpack aspirator mosquito collections (January 2006–January 2007) near control blocks

**Figure 3:**
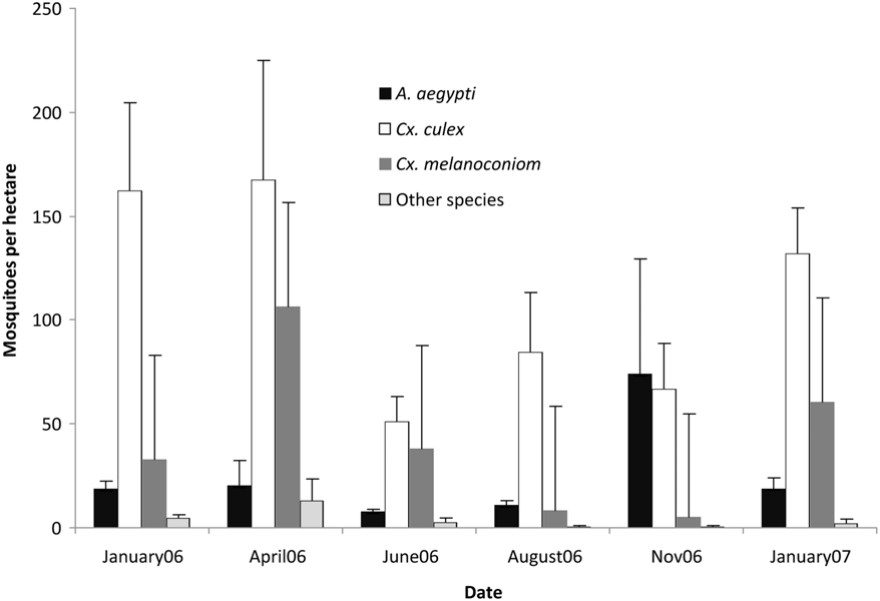
Population densities of mosquitoes collected during household entomological surveys. Mosquitoes were collected with backpack aspirators in house-to-house daytime surveys, carried out in 50 blocks distributed among 10 Ministry of Health zones. Results are represented as the mean (±std error) of the number of mosquitoes collected per hectare surveyed. The other species category includes mosquitoes from the following genera: *Aedomyia*, *Coquillitidea, Psorophora, Uranotaenia, Mansonia, Ochlerotatus,* and *Wyeomia.*

As part of ongoing studies of dengue vector epidemiology, our NMRCD/UC Davis study team carried out standardized *Aedes aegypti* surveys at approximately two-month intervals on 3–5 blocks in 10 geographic zones. Mosquitoes captured during backpack aspirator collection were identified to genera. *Culex* species from both the *Culex* and *Melanoconion* sub*-*genera were commonly collected inside houses during the day. In addition, specimens from the genera *Psorophora*, *Uranotaenia, Mansonia,* and *Ochlerotatus* were commonly observed. The number of *Culex (Cx.)* spp. ranged from 2–675 adults per hectare surveyed, whereas *Culex (Mel.)* spp. were less abundant, ranging from 2–246 adults per hectare (Figure 3). Abundance levels showed both geographic and temporal differences; most notably *Culex (Mel.)* spp. population levels appeared highest during our April 2006 surveys and were clearly highest during the first half of the year (Figure 3). These collections were not designed to monitor population abundance of these species in a systematic way, rather to illustrate that these species are present and can reach high population densities in urban areas of Iquitos. No routine surveys were carried out the Bella Vista Nanay, San Juan, or Belen areas during the first half of 2006 when VEEV activity was detected in the febrile surveillance program. Between February 17 and March 17, routine collections were carried out in the same MOH zone as the Belen neighborhood (Figure 1); 152 *Culex (Cx.) spp.,* 13 *Culex (Mel) spp.,* and 8 other non-*Aedes* species were observed per hectare surveyed.

#### Post-outbreak mosquito collections

To more systematically address the presence of potential VEEV vectors in neighborhoods with high numbers of VEEV cases, in October 2006 both household and CDC light-trap surveys were conducted on the blocks corresponding to the serological survey. Overall, mosquito abundance levels were consistent with those observed in other parts of the city at that time of year, but were lower than those observed during the first half of 2006 (Table 7).

**Table 7:** Number of adult mosquitoes collected per hectare in house-to-house backpack aspirator collections carried out in October 2006.

Neighborhood	Culex (Cx)	Culex (Mel)	Psorophora	Uranotaenia	Mansonia	Others
**Belen**	**28**	**1.28**	**0.0**	**0.0**	**0.0**	**0.0**
**Bella Vista Nanay**	**124.62**	**0.79**	**0.0**	**0.52**	**0.0**	**0.0**
San Pedro	8.85	0.0	0.0	0.0	0.0	0.0
Nuevo Bellavista	144.71	0.92	0.0	0.61	0.0	0.0
**San Juan**	**233.7**	**15.76**	**2.49**	**3.11**	**0.62**	**0.21**
Las Mercedes	318.08	10.12	6.74	1.12	0.00	0.56
San Pablo	162.29	20.39	0.0	5.52	1.27	0.00
26 de Febrero	259.72	14.51	0.0	0.00	0.00	0.00

A total of 12,065 mosquitoes were collected from 208 CDC light trap-nights baited with CO_2_ during September and October. Genera collected include *Aedeomyia, Aedes, Anopheles, Coquillettidia, Culex, Mansonia, Ochlerotatus,* and *Uranotaenia* (Table 8). There was an average of 58 mosquitoes per trap-night observed in the five neighborhoods sampled. The highest number captured (160 mosquitoes/trap-night) was in the neighborhood 26 de Febrero located on the Avenue La Participación, near the Iquitos-Nauta Highway on the edge of urban Iquitos. The lowest number (1.7 mosquitoes/trap-night) was captured in Belen. The other neighborhoods located in San Juan, Las Mercedes and San Pablo de la Luz had intermediate mosquito densities of 47 mosquitoes per trap-night. Finally, in Bella Vista Nanay 17 mosquitoes were captured per trap-night. The most common species observed (72% of total captured mosquitoes) in each of the sampled neighborhoods was *Culex quinquefasciatus*, a cosmopolitan species with a worldwide distribution, especially in urban areas. In this study, 1,276 specimens (11%) were from the *Culex (Mel.)* group. *Culex (Mel.) ocossa* was the most abundant *Melanoconion* species previously implicated as a principal VEE vector, whereas *Psophora cingulata and Mansonia indubitans/tillillans* were the most abundant secondary vectors observed [40],[41]. All female mosquitoes collected were homogenized and tested for VEEV RNA by RT-PCR. All specimens tested were negative.

**Table 8:** Mosquitoes collected in CDC Light traps in neighborhoods with clinical cases of VEEV infection in 2006.

Species	Bella Vista Nanay	San Juan (Av. La Participación)			Belén	Total
		Las Mercedes	San Pablo de Luz	26 de Febrero		
*Ad. (Ady.) squamipennis*	1	1	1	2	0	5
*Ae. (Stg.) aegypti*	0	0	7	3	0	10
*An. (Nys.) sp*	0	0	0	1	0	1
*An. (Nys.) triannulatus*	0	1	0	0	0	1
*Cq. (Rhy.) hermanoi*	0	0	1	1	0	2
*Cq. (Rhy.) nigricans*	0	1	5	7	0	13
*Cq. (Rhy.) venezuelensis*	2	19	7	43	0	71
*Cx. (Ads.) amazonensis*	5	0	18	45	0	68
*Cx. (Cx.) corniger*	0	0	0	0	1	1
*Cx. (Phe.) corniger*	7	0	0	2	1	10
*Cx. (Cx.) coronator*	17	26	13	27	4	87
*Cx. (Cx.) declarator/mollis*	27	157	126	28	2	340
Cx. (Cx.) quinquefasciatus	255	1054	1531	5786	45	8671
*Cx. (Cx.)* sp	18	90	178	589	1	876
***Cx. (Mel.) adamesi *** [***22***]	0	0	1	1	0	2
*Cx. (Mel.) atratus* Group	0	0	1	0	0	1
*Cx. (Mel.) declarator/mollis*	0	0	2	0	0	2
*Cx. (Mel.) dunni*	0	0	3	0	0	3
***Cx. (Mel.) gnomatus *** [***40***],[***43***]	0	0	0	1	0	1
***Cx. (Mel.) ocossa *** [***41***],[***22***]	56	12	86	687	1	842
***Cx. (Mel.) pedroi *** [***40***],[***22***]	1	1	3	18	1	24
***Cx. (Mel.) portesi *** [***41***],[***22***]	1	0	3	12	0	16
*Cx. (Mel.)* sp*	49	29	74	163	14	329
***Cx. (Mel.) spissipes *** [***22***]	0	0	1	0	0	1
*Cx. (Mel.) theobaldi*	2	4	10	0	0	16
***Cx. (Mel.) vomerifer*** [***40]***,[***22***]	5	2	13	17	2	39
*Ma. (Man.) amazonensis*	3	11	0	0	0	14
*Ma. (Man.) flaveola*	0	0	0	0	1	1
*Ma. (Man.) humeralis*	19	0	0	1	0	20
***Ma. (Man.) indubitans/titillans *** [***40***]	48	20	26	104	2	200
*Ma. (Man.) pseudotitillans*	1	1	0	1	0	3
*Oc. (Och.) fulvus*	1	0	0	0	0	1
*Oc. (Och.) hastatus*	0	0	1	0	0	1
***Ps. (Gra.) cingulata *** [***40***]	1	58	102	58	0	219
***Ps. (Jan.) albigenu *** [***40***]	6	0	0	1	0	7
*Ur. (Ura.) apicalis*	2	0	3	14	1	20
*Ur. (Ura.) gemetrica*	0	1	0	2	0	3
*Ur. (Ura.) hystera*	0	1	44	46	2	93
*Ur. (Ura.) iowii*	29	1	4	1	6	41
*Ur. (Ura.) pallidoventer*	0	0	7	1	1	9
*Ur. (Ura.) sp*	0	0	0	1	0	1

Citations are provided for species previously implicated as vectors (shown in **bold**).

*: Undescribed species for Peruvian Amazon.

## Discussion

Based on data from a clinic-based febrile illness surveillance program, transmission of enzootic VEEV subtypes has been well documented in the Iquitos area of northeastern Peru at consistent but low levels since the early 1990s [6],[19],[42]. In this study we report an outbreak of human VEEV infections during the first half 2006 detected through the NMRCD surveillance program, with the majority of patients residing within city limits. In response to this outbreak, we conducted an antibody prevalence study and mosquito collections within urban areas of Iquitos, targeting neighborhoods with large numbers of cases during the 2006 outbreak. The prevalence of VEEV antibody exceeded 18% in all areas, and known vectors of the disease were identified across the city. To our knowledge, this is the first antibody survey for enzootic VEEV in an urban population.

Enzootic subtypes of VEEV have been thought to be maintained primarily in sylvatic cycles of tropical and sub-tropical forests. Consistent with this idea, we found acute VEEV infection to be more common among febrile patients residing outside of Iquitos, adjacent to the rain forest. Furthermore, we found multi-day travel and forest-related occupations to be statistically significant risk factors for VEEV antibody positivity. However, while transmission may be higher in rural areas, several lines of evidence suggest that transmission occurs within the urban areas of Iquitos as well. First, while forest-related occupation was a significant risk factor, these occupations comprised only a very small percentage of the total. In previous studies, enzootic VEEV clinical disease has been most frequently reported in adult males due to this association with high-risk forest occupations [43]. In contrast, we found no correlation in this study between gender and VEEV antibody status or acute VEEV infection. Second, consistent with prior reports [6], the majority of the acute human cases detected in the passive surveillance program maintained residence within the city proper and did not report recent travel. Third, nearly 70% of VEEV-antibody positive survey respondents did not report recent travel, and day trips were not strongly associated with antibody positivity. It should be noted that our study might underestimate respondents' exposure to the forested zones surrounding Iquitos, as recall bias is likely a limitation in obtaining accurate travel history information. Furthermore, our antibody prevalence study did not adequately control for migration into the city. Specifically, we did not determine the length of residence at the current address for study participants or the location of previous residence. Especially in older age groups, infection before establishing residence in Iquitos must be considered. Despite these limitations, the existing information suggests that some level of VEEV transmission occurs within urban Iquitos.

While our data suggest that enzootic VEEV strains are transmitted in urban areas, the exact mechanism is unclear. One possibility is the existence of a self-perpetuating endemic cycle established within urban Iquitos. Alternatively, urban cases of enzootic VEEV may be caused by repeated introductions of the virus from local forests, either by infected vectors or by infected hosts. Enzootic VEEV has been isolated from spiny rats (*Proechimys* spp.) in the region [6]; however, within the city limits of Iquitos rodent fauna appears to be mostly limited to *Rattus ratus*, *Rattus norvegicus* and *mus musculus* [44]. Iquitos is geographically isolated within the Amazon forest, and the distances between natural forest cycles and the city may well be within the natural home range of vectors and reservoirs. Within 20 km of the city there is a diversity of mammalian fauna, including rodents (*Proechimys* spp., *Oryzomys* spp., *Neacomys* spp. , and *Dasyprocia* spp.), marsupials (*Phliander* spp., *Marmosops* spp., *Micoureus* spp., *Caluromys* spp., *Metachirus* spp., and *Monodelphis* spp.), bats (*Platyrihinus* spp., *Artibeius* spp., *Sturnira* spp., and *Carollia* spp.) and sloths (*Choloepus* spp. and *Bradypus* spp.) In addition to sylvatic rodents, various species of waterfowl are readily infected by enzootic VEEV [41],[45]. Such birds, if found to be amplifying reservoirs, could quickly expand the geographic and ecological distribution of the virus. Delineating the precise mechanism of urban transmission will require identification of relevant vectors and reservoir hosts currently infected and circulating within the city.

Mosquitoes from the *Culex (Mel.)* species have been previously implicated as the primary vectors of enzootic subtypes of VEEV [40],[46],[47]. In this study, we found that *Culex (Mel.)* species are present throughout the city and abundant at certain times of the year. Most notably, *Cx. (Mel.) ocossa,* the vector of ID VEEV in Panama [41], was observed in significant numbers. We have also identified the presence of other genera within urban Iquitos that have been previously incriminated as potential vectors for enzootic VEEV. For example, in our study blocks we detected *Psorophora* (especially *cingulata*), *Mansonia* and *Coquillettidia* species, which have been shown to be competent vectors in the laboratory [48],[49]. Additionally, *Aedes aegypti* has been shown to be a competent vector of both enzootic [50] and epizootic VEEV in the laboratory [51],[52],[53],[54] and is present throughout the city of Iquitos. While feeding preference, as well as temporal and spatial distribution, argues against a role for *Aedes aegypti* in VEEV transmission cycles in Iquitos, the possibility warrants further examination. In the current study, we tested a wide range of mosquito species collected from CDC traps for the presence of VEEV RNA. We were unable to detect VEEV in any of the mosquito species tested; however, these studies were conducted at least four months after the last 2006 case was detected in the passive surveillance study. Furthermore, in previous studies VEEV infection rates in mosquitoes have been found to be very low [16],[20]. Turell et al., for example, recovered 25 VEEV isolates from 245,053 *Culex (Mel.)* spp. specimens [20]. In that study, 14 of 25 VEEV isolates were from *Culex (Mel.) gnomatos*; in our study, only one *Culex (Mel.) gnomatos* specimen was collected. To clearly define urban transmission patterns within Iquitos, prospective studies of potential vectors, including VEEV isolation and abundance pattern characterization, are needed during seasons of high incidence.

Irrespective of species, there is compelling indirect evidence linking mosquito exposure within the city to prior VEEV infection. First, Bella Vista Nanay and San Jaun had higher mosquito abundance in both household surveys and CDC light-trap collections than the two neighborhoods with lower seroprevalence rates. Furthermore, mosquito net use was significantly more common in neighborhoods with higher seroprevalence than in those with lower seroprevalence rates. The fact that mosquito-net use was a risk factor for previous infection VEEV may seem counterintuitive, but it is also a proxy for intensity of exposure to mosquito bites. In areas where biting intensity is higher more individuals use nets out of necessity. This association needs to be interpreted in the context of the specifics of mosquito net use (eg. the precise time of day that are people protected) and condition. Many respondents mentioned bathing in the river at sunset; *Culex* mosquitoes are crepuscular and thus would have access to people during the dusk hours regardless of nighttime mosquito net use. Overall, it is clear that exposure to potential mosquito vectors occurs in all areas of the city, but this evidence indicates that exposure is highest in the areas with the highest seropositivity.

The cause for the 2006 outbreak of VEEV infections is unclear. It is interesting to note that the 2006 outbreak inside the city was preceded by a spike in cases just outside the city in 2005. There are several possibilities that might explain both increases. Vector abundance may have increased due to cyclical weather patterns, increasing rates of transmission in the forest, with a subsequent urban spillover. Furthermore unusually high annual river levels occurring in early 2006 may have increased competent vectors within the city (i.e., *Cx. (Mel.) ocossa*) leading to the observed urban VEEV cases. Alternatively, human encroachment on forest areas, due to activities such as agriculture and logging, may have increased human contact with established enzootic transmission cycles and altered vector and reservoir distribution. Mutations in circulating sylvatic viruses might have led to greater potential for human infection and disease, and thus an increase in clinical cases. In light of the genetic similarity between enzootic and epizootic strains of VEEV, the possibility of humans as productive hosts, as well as the potential for emergence of an epidemic strain in urban areas, needs to be considered.

## Supporting Information

Figure S1 Temperature, rainfall, and river levels for Iquitos, Peru 2000–2006.
(0.41 MB TIF)

Figure S2 Phylogenetic tree depicting evolutionary relationships between members of the Venezuelan equine encephalitis virus complex, based on partial sequences of the PE2 gene. The depicted tree is based on the neighbor-joining (shown) and maximum parsimony analyses, implemented in Mega4 (Tamura et al., 2007). Bootstrap support values for the respective clades are indicated to the left. Viral strains are indicated by VEEV complex subtype, country abbreviation and year of isolation, followed by the sample code. Selected viral isolates collected from febrile patients in Iquitos during 2006 are indicated in bold.
(0.05 MB PDF)

Table S1 Inspection of Vegetation in Bellavista Nanay and San Juan Study Neighborhoods.
(0.07 MB DOC)

## References

[1] Kubes V, R¡os FA (1939). The causative agent of infectious equine encephalomyelitis in venezuela.. Science.

[2] Kubes V, Gallia F (1944). Neutralization of Encephalomyelitis Virus by Human Sera.. Canadian journal of comparative medicine and veterinary science.

[3] Lord RD (1974). History and geographic distribution of Venezuelan equine encephalitis.. Bulletin of the Pan American Health Organization.

[4] Weaver SC, Barrett AD (2004). Transmission cycles, host range, evolution and emergence of arboviral disease.. Nat Rev Microbiol.

[5] Johnson KM, Martin DH (1974). Venezuelan equine encephalitis.. Advances in veterinary science and comparative medicine.

[6] Aguilar PV, Greene IP, Coffey LL, Medina G, Moncayo AC (2004). Endemic Venezuelan equine encephalitis in northern Peru.. Emerg Infect Dis.

[7] Estrada-Franco JG, Navarro-Lopez R, Freier JE, Cordova D, Clements T (2004). Venezuelan equine encephalitis virus, southern Mexico.. Emerg Infect Dis.

[8] Oberste MS, Schmura SM, Weaver SC, Smith JF (1999). Geographic distribution of Venezuelan equine encephalitis virus subtype IE genotypes in Central America and Mexico.. Am J Trop Med Hyg.

[9] Oberste MS, Fraire M, Navarro R, Zepeda C, Zarate ML (1998). Association of Venezuelan equine encephalitis virus subtype IE with two equine epizootics in Mexico.. Am J Trop Med Hyg.

[10] Anishchenko M, Bowen RA, Paessler S, Austgen L, Greene IP (2006). Venezuelan encephalitis emergence mediated by a phylogenetically predicted viral mutation.. Proc Natl Acad Sci U S A.

[11] Powers AM, Oberste MS, Brault AC, Rico-Hesse R, Schmura SM (1997). Repeated emergence of epidemic/epizootic Venezuelan equine encephalitis from a single genotype of enzootic subtype ID virus.. Journal of virology.

[12] Wang E, Barrera R, Boshell J, Ferro C, Freier JE (1999). Genetic and phenotypic changes accompanying the emergence of epizootic subtype IC Venezuelan equine encephalitis viruses from an enzootic subtype ID progenitor.. Journal of virology.

[13] Weaver SC, Bellew LA, Rico-Hesse R (1992). Phylogenetic analysis of alphaviruses in the Venezuelan equine encephalitis complex and identification of the source of epizootic viruses.. Virology.

[14] Weaver SC, Salas R, Rico-Hesse R, Ludwig GV, Oberste MS (1996). Re-emergence of epidemic Venezuelan equine encephalomyelitis in South America. VEE Study Group.. Lancet.

[15] Weaver SC, Anishchenko M, Bowen R, Brault AC, Estrada-Franco JG (2004). Genetic determinants of Venezuelan equine encephalitis emergence.. Arch Virol.

[16] Scherer WF, Madalengoitia J, Menesis O, Acosta M (1979). Study of VE virus and isolation of SLE, EE, group C, and GUAMA group arboviruses in the Amazon region of Peru, 1975.. Bull Pan Am Health Organ.

[17] Scherer WF, Madalengoitia J, Flores W, Acosta M (1975). Ecologic studies of Venezuelan encephalitis virus in Peru during 1970–1971.. American journal of epidemiology.

[18] Watts DM, Lavera V, Callahan J, Rossi C, Oberste MS (1997). Venezuelan equine encephalitis and Oropouche virus infections among Peruvian army troops in the Amazon region of Peru.. The American journal of tropical medicine and hygiene.

[19] Watts DM, Callahan J, Rossi C, Oberste MS, Roehrig JT (1998). Venezuelan equine encephalitis febrile cases among humans in the Peruvian Amazon River region.. The American journal of tropical medicine and hygiene.

[20] Turell MJ, O'Guinn ML, Jones JW, Sardelis MR, Dohm DJ (2005). Isolation of viruses from mosquitoes (Diptera: Culicidae) collected in the Amazon Basin region of Peru.. Journal of medical entomology.

[21] Galindo P, Grayson MA (1971). Culex (Melanoconion) aikenii: natural vector in Panama of endemic Venezuelan encephalitis.. Science (New York, NY).

[22] Ferro C, Boshell J, Moncayo AC, Gonzalez M, Ahumada ML (2003). Natural enzootic vectors of Venezuelan equine encephalitis virus, Magdalena Valley, Colombia.. Emerging infectious diseases.

[23] Turell MJ, Barth J, Coleman RE (1999). Potential for Central American mosquitoes to transmit epizootic and enzootic strains of Venezuelan equine encephalitis virus.. Journal of the American Mosquito Control Association.

[24] Morrison AC, Astete H, Chapilliquen F, Ramirez-Prada C, Diaz G (2004). Evaluation of a sampling methodology for rapid assessment of Aedes aegypti infestation levels in Iquitos, Peru.. Journal of medical entomology.

[25] Morrison AC, Gray K, Getis A, Astete H, Sihuincha M (2004). Temporal and geographic patterns of Aedes aegypti (Diptera: Culicidae) production in Iquitos, Peru.. Journal of medical entomology.

[26] Getis A, Morrison AC, Gray K, Scott TW (2003). Characteristics of the spatial pattern of the dengue vector, Aedes aegypti, in Iquitos, Peru.. The American journal of tropical medicine and hygiene.

[27] Hayes CG, Phillips IA, Callahan JD, Griebenow WF, Hyams KC (1996). The epidemiology of dengue virus infection among urban, jungle, and rural populations in the Amazon region of Peru.. The American journal of tropical medicine and hygiene.

[28] Schneider JR, Morrison AC, Astete H, Scott TW, Wilson ML (2004). Adult size and distribution of Aedes aegypti (Diptera: Culicidae) associated with larval habitats in Iquitos, Peru.. Journal of medical entomology.

[29] ODEI-Loreto (2006). Censos Nacionales 2005: X de Población y V de Vivienda. Boletin Informativo….

[30] Bunnell JE, Hice CL, Watts DM, Montrueil V, Tesh RB (2000). Detection of pathogenic Leptospira spp. infections among mammals captured in the Peruvian Amazon basin region.. Am J Trop Med Hyg.

[31] Jones JW, Turell MJ, Sardelis MR, Watts DM, Coleman RE (2004). Seasonal distribution, biology, and human attraction patterns of culicine mosquitoes (Diptera: Culicidae) in a forest near Puerto Almendras, Iquitos, Peru.. J Med Entomol.

[32] Consoli GB, de Oliveira RL (1994). Principias mosquitos de importancia sanitaria no brasil rotrauta. 1994: FIOCRUZ..

[33] Caceda ER, Kochel TJ (2007). Application of modified shell vial culture procedure for arbovirus detection.. PLoS ONE.

[34] Oberste MS, Weaver SC, Watts DM, Smith JF (1998). Identification and genetic analysis of Panama-genotype Venezuelan equine encephalitis virus subtype ID in Peru.. Am J Trop Med Hyg.

[35] Ansari MZ, Shope RE, Malik S (1993). Evaluation of vero cell lysate antigen for the ELISA of flaviviruses.. Journal of clinical laboratory analysis.

[36] Innis BL, Nisalak A, Nimmannitya S, Kusalerdchariya S, Chongswasdi V (1989). An enzyme-linked immunosorbent assay to characterize dengue infections where dengue and Japanese encephalitis co-circulate.. The American journal of tropical medicine and hygiene.

[37] Morens DM, Halstead SB, Repik PM, Putvatana R, Raybourne N (1985). Simplified plaque reduction neutralization assay for dengue viruses by semimicro methods in BHK-21 cells: comparison of the BHK suspension test with standard plaque reduction neutralization.. J Clin Microbiol.

[38] Aguilar PV, Robich RM, Turell MJ, O'Guinn ML, Klein TA (2007). Endemic eastern equine encephalitis in the Amazon region of Peru.. Am J Trop Med Hyg.

[39] Lanciotti RS, Kerst AJ, Nasci RS, Godsey MS, Mitchell CJ (2000). Rapid detection of west nile virus from human clinical specimens, field-collected mosquitoes, and avian samples by a TaqMan reverse transcriptase-PCR assay.. Journal of clinical microbiology.

[40] Turell MJ, Jones JW, Sardelis MR, Dohm DJ, Coleman RE (2000). Vector competence of Peruvian mosquitoes (Diptera: Culicidae) for epizootic and enzootic strains of Venezuelan equine encephalomyelitis virus.. Journal of medical entomology.

[41] Galindo P (1972). Endemic vectors of Venezuelan encephalitis.. Proceedings of Workshop-Symposium on Venezuelan Encephalitis Virus, Washington, DC, 14–17 September 1971.

[42] Yanoviak SP, Aguilar PV, Lounibos LP, Weaver SC (2005). Transmission of a Venezuelan equine encephalitis complex Alphavirus by Culex (Melanoconion) gnomatos (Diptera: Culicidae) in northeastern Peru.. J Med Entomol.

[43] Walton TE, Grayson MA (1988). Venezuelan equine encephalomyelitis. The Arboviruses: Epidemiology and Ecology.

[44] Johnson MA, Smith H, Joeph P, Gilman RH, Bautista CT (2004). Environmental exposure and leptospirosis, Peru.. Emerg Infect Dis.

[45] Dickerman RW, Bonacorsa CM, Scherer WF (1976). Viremia in young herons and ibis infected with Venezuelan encephalitis virus.. Am J Epidemiol.

[46] Cupp EW, Scherer WF, Ordonez JV (1979). Transmission of Venezuelan encephalitis virus by naturally infected Culex (Melanoconion) opisthopus.. Am J Trop Med Hyg.

[47] Turell MJ (1999). Vector competence of three Venezuelan mosquitoes (Diptera: Culicidae) for an epizootic IC strain of Venezuelan equine encephalitis virus.. Journal of medical entomology.

[48] Ortiz DI, Anishchenko M, Weaver SC (2005). Susceptibility of Psorophora confinnis (Diptera: Culicidae) to infection with epizootic (subtype IC) and enzootic (subtype ID) Venezuelan Equine encephalitis viruses.. J Med Entomol.

[49] Moncayo AC, Lanzaro G, Kang W, Orozco A, Ulloa A (2008). Vector competence of eastern and western forms of Psorophora columbiae (Diptera: Culicidae) mosquitoes for enzootic and epizootic Venezuelan equine encephalitis virus.. Am J Trop Med Hyg.

[50] Coffey LL, Vasilakis N, Brault AC, Powers AM, Tripet F (2008). Arbovirus evolution in vivo is constrained by host alternation.. Proc Natl Acad Sci U S A.

[51] Mellink JJ (1982). Estimation of the amount of Venezuelan equine encephalomyelitis virus transmitted by a single infected Aedes aegypti (Diptera: Culicidae).. Journal of medical entomology.

[52] Mellink JJ (1982). Transmission of Venezuelan equine encephalomyelitis virus by Aedes aegypti (Diptera: Culicidae) to mice previously exposed to vector antigens.. Journal of medical entomology.

[53] Gaidamovich SY, Tsilinsky YY, Lvova AI, Khutoretskaya NV (1971). Aedes aegypti mosquitoes as an experimental model for studies on the ecology and genetics of Venezuelan equine encephalomyelitis virus.. Acta virologica.

[54] Woodward TM, Miller BR, Beaty BJ, Trent DW, Roehrig JT (1991). A single amino acid change in the E2 glycoprotein of Venezuelan equine encephalitis virus affects replication and dissemination in Aedes aegypti mosquitoes.. The Journal of general virology.

