# Supplementary figures and images for: Venezuelan Equine Encephalitis Virus in Iquitos, Peru: Urban Transmission of a Sylvatic Strain
Source: PLoS Negl Trop Dis. 2008 Dec 16;2(12):e349. doi: 10.1371/journal.pntd.0000349 (PMC2593782; doi:10.1371/journal.pntd.0000349)

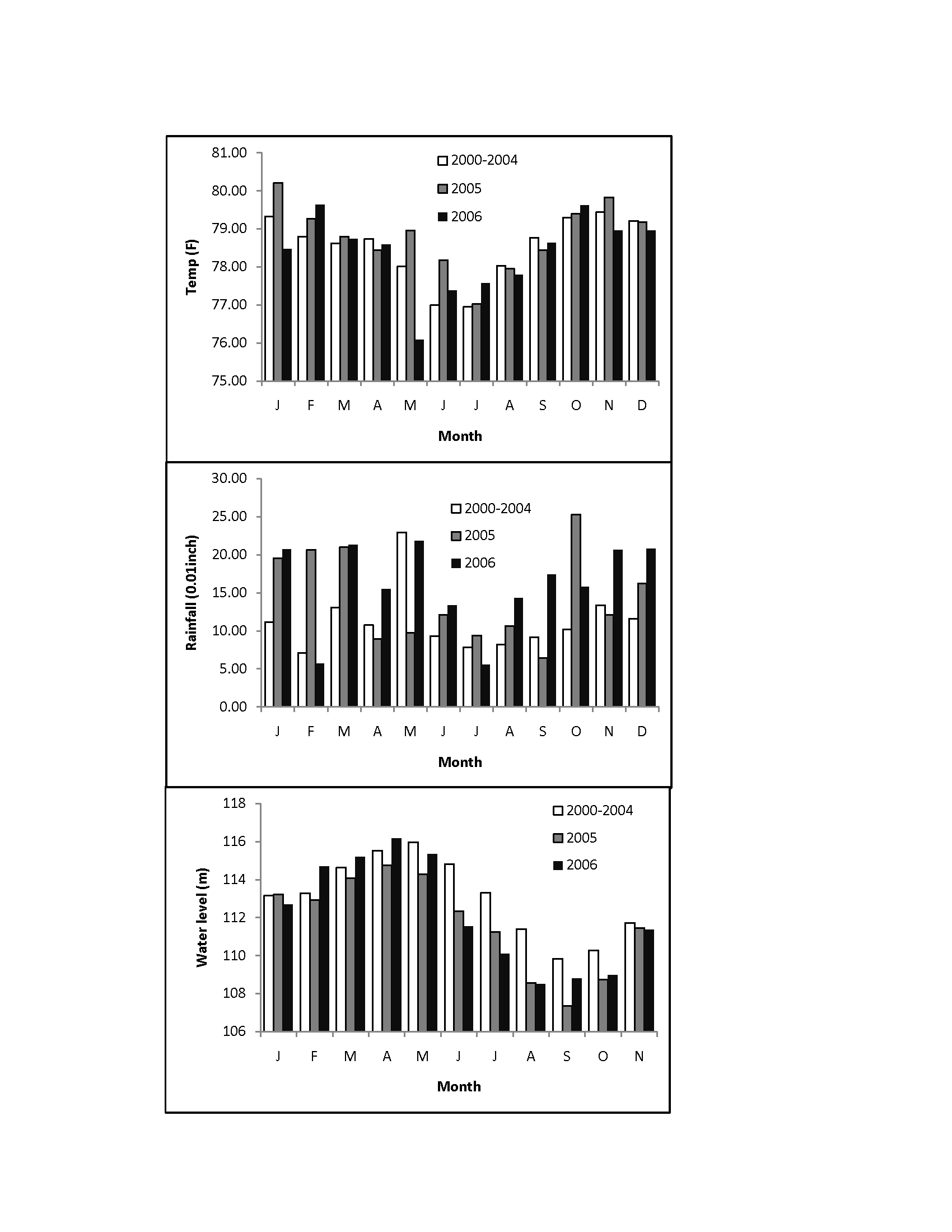

Supplement: Figure S1 — Temperature, rainfall, and river levels for Iquitos, Peru 2000–2006. (0.41 MB TIF) [file pntd.0000349.s001.tif]

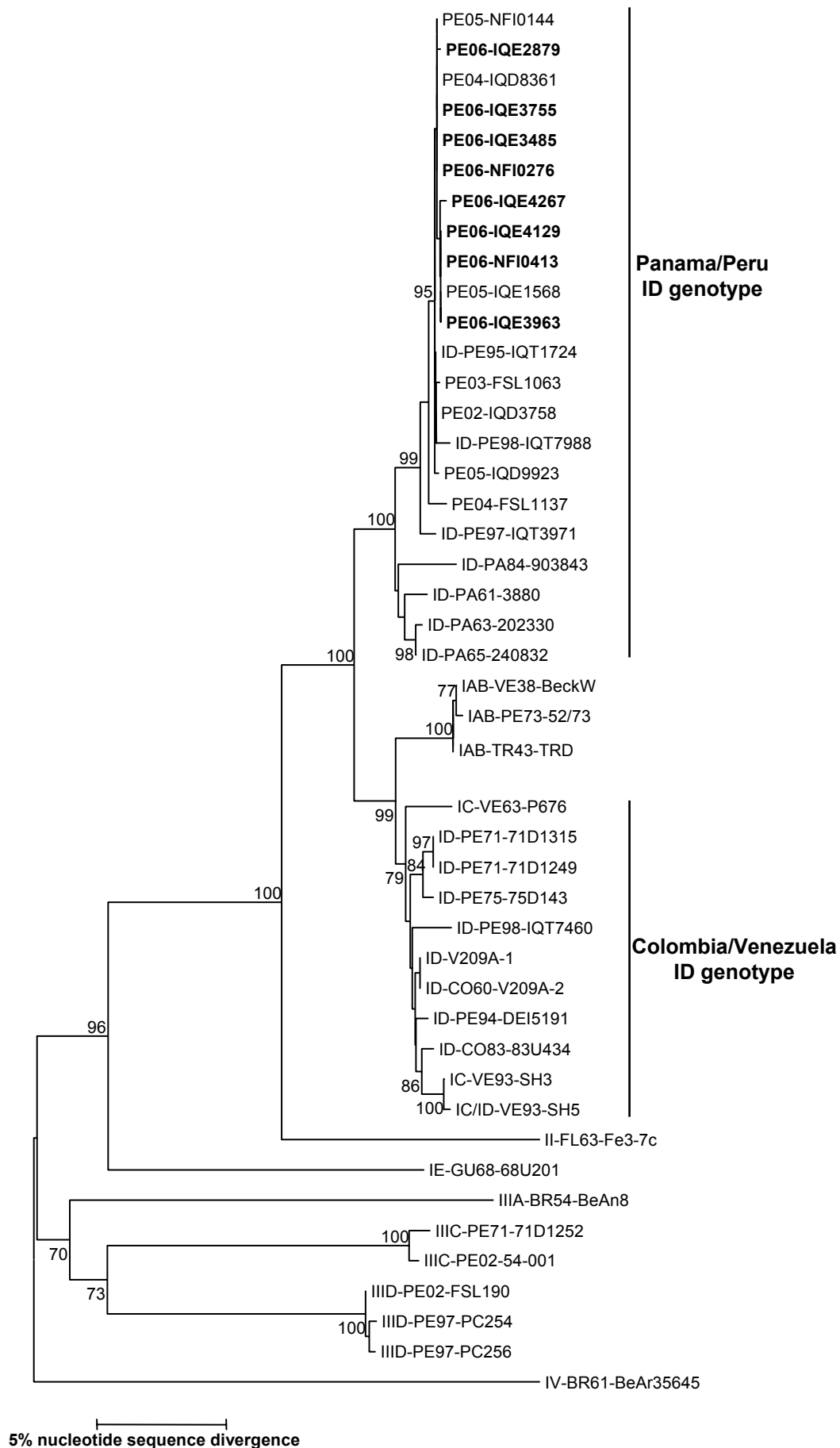

Supplement: Figure S2 — Phylogenetic tree depicting evolutionary relationships between members of the Venezuelan equine encephalitis virus complex, based on partial sequences of the PE2 gene. The depicted tree is based on the neighbor-joining (shown) and maximum parsimony analyses, implemented in Mega4 (Tamura et al., 2007). Bootstrap support values for the respective clades are indicated to the left. Viral strains are indicated by VEEV complex subtype, country abbreviation and year of isolation, followed by the sample code. Selected viral isolates collected from febrile patients in Iquitos during 2006 are indicated in bold. (0.05 MB PDF) [file pntd.0000349.s002.pdf]
