# Supplementary material for: Venezuelan Equine Encephalitis Virus in Iquitos, Peru: Urban Transmission of a Sylvatic Strain
Source: PLoS Negl Trop Dis. 2008 Dec 16;2(12):e349. doi: 10.1371/journal.pntd.0000349 (PMC2593782; doi:10.1371/journal.pntd.0000349)
Supplement: Table S1 — Inspection of Vegetation in Bellavista Nanay and San Juan Study Neighborhoods. (0.07 MB DOC) [file pntd.0000349.s003.doc]

**Inspection of Vegetation of Bellavista Nanay and San Juan**

Date: August 1, 2008.

Description of vegetation present.

1. Bellavista Nanay

Area with seasonal flooding by the Nanay River (black wáter).

| **Familia** | **Species** | **Local Name** |
| --- | --- | --- |
|
| Polygonaceae | Triplaris sp. | Tangarana |
| Lauraceae | Ocotea sp. | Moena |
| Fabaceae | Erythyna sp. | Amasisa |
| Cecropiaceae | Cecropia sp. | Cetico |
| Fabaceae | Senna sp. | Retama |
| Myrtaceae | Myrciaria sp. | Camu camu |
| Poaceae | *Paspalum repens* | Gramalote |
| Pontederiaceae | *Eichornia crasipes* | Putu putu |
| Polygonaceae | *Polygon ferrugineum* | Tabaco Lagarto |
| Acanthaceae | *Justicia* sp. |  |
| Poaceae | *Panicum* sp. | Grama |
| Onagraceae | *Ludwigia* sp. |  |
| Pontederiaceae | *Eichornia azurea* | Putu putu |
| Poaceae | *Gynerium sagitattum* | Caña brava |
| Onagraceae | *Ludwigia sp.* |  |
| Araceae | *Pistia stratiotes* | Huama |

Observations: Trees and bushes were dispersed, some species were concentrated in near the edge of wáter bodies, the rest of the species were distributed randomly. The aquatic plants were concentrated in colonies.

1. San Juan (Av. Participacion)

Area with seasonal flooding by the River Itaya (white wáter)

| **Familia** | **Species** | **Local Name** |
| --- | --- | --- |
|
| Moraceae | *Ficus antihelmitica* | Oje |
| Polygonacea | *Triplaris* sp | Tangarana |
| Fabaceae | *Senna reticulata* | Retama |
| Cecropiaceae | Cecropia sp | Cetico |
| Euphorbiaceae | *Hura crepitans* | Catahua |
| Bombaceae | *Chlorisia sp* | Lupuna |
| Euphorbiaceae | *Aparisthmium* sp |  |
| Anacardiaceae | *Tapirira* sp |  |
| Fabaceae | *Swartzia* sp |  |
| Polygonaceae | *Coccoloba* sp |  |
| Bombaceae | *Pachira* sp | Punga |
| Arecacea | *Bactris* sp | Nejilla |
| Arecacea | *Astrocalyum* sp |  |
| Poaceae | *Paspalum repens* | Gramalote |
| Cyperaceae | *Cyperus* sp |  |
| Malvaceae |  |  |
| Bromelaceae | *Tilandsia* sp | Piña |
| Orchidaceae | *Octomeria* sp |  |
| Asteraceae | *Clidemia* sp |  |
| Zingiberaceae | *Costus* sp |  |
| Poaceae | *Oryza* sp |  |
| Solanaceae | *Solanum* sp |  |
| Verbenaceae | *Lantana lamosa* |  |
| Malvaceae | *Urena lobata* |  |
| Poaceae | *Panicum* sp |  |
| Onagraceae | *Ludwigia* sp |  |
| Malvaceae | *Sida* sp |  |
| Asteraceae | *Vernomia* sp |  |
| Melastomataceae | *Tococa* sp |  |
| Arecaceae | *Bactris* sp |  |
| Arecacea | *Astrocaryum* sp |  |
| Passifloraceae | *Passiflora foetida* |  |
| Vitaceae | *Cissus erosa* |  |
| Arecaceae | *Descomcus* sp |  |
| Fabaceae | *Enterolobium* sp |  |
| Loganiaceae | *Strichnos* sp |  |

Observations: Trees and bushes are more grouped within the area apparently with more species diversity and density than in other areas.
